# Interface Adhesion and Structural Characterization of Rolled-up GaAs/In_0.2_Ga_0.8_As Multilayer Tubes by Coherent Phonon Spectroscopy

**DOI:** 10.1038/s41598-017-05739-6

**Published:** 2017-07-14

**Authors:** D. Brick, V. Engemaier, Y. Guo, M. Grossmann, G. Li, D. Grimm, O. G. Schmidt, M. Schubert, V. E. Gusev, M. Hettich, T. Dekorsy

**Affiliations:** 1 Department of Physics, University of Konstanz, 78464 Konstanz, Germany; 2 Institute for Integrative Nanosciences, IFW Dresden, Helmholtzstrasse 20, 01069 Dresden, Germany; 3 LAUM, UMR-CNRS 6613, Université du Maine, Av. O. Messiaen, 72085 Le Mans, France; 4 Institute of Technical Physics, German Aerospace Center, Pfaffenwaldring 38-40, 70569 Stuttgart, Germany

## Abstract

We present a detailed experimental and theoretical study of the acoustic phonon modes in rolled-up multilayers with thickness of the layers in the nanometre and diameters in the micrometre range. We compare our results to planar, unrolled multilayers grown by molecular beam epitaxy. For the planar multilayers the experimentally obtained acoustic modes exhibit properties of a superlattice and match well to calculations obtained by the Rytov model. The rolled-up superlattice tubes show intriguing differences compared to the planar structures which can be attributed to the imperfect adhesion between individual tube windings. A transfer matrix method including a massless spring accounting for the imperfect adhesion between the layers yields good agreement between experiment and calculations for up to five windings. Areas with sufficient mechanical coupling between all windings can be distinguished by their acoustic mode spectrum from areas where individual windings are only partially in contact. This allows the spatially resolved characterization of individual tubes with micrometre spatial resolution where areas with varying interface adhesion can be identified.

## Introduction

Rolled-up microtubes^1, 2^ are promising candidates for applications in various interdisciplinary fields such as in microfluidics^3^, biophysics^4^, and in optoelectronics^5^. The fabrication technique of these three-dimensional structures also provides a new pathway for the realization of hybrid radial superlattices^6–9^. Potential applications are thermoelectric devices^10^, field effect transistors^3^ and metamaterial optical hyperlens structures^11^. Not only the radial geometry of the structures itself but also the simplicity of the fabrication process^1, 2^ compared to conventional techniques in addition with the possibility to use novel materials and material combinations^6, 12^ which remain challenging for usual growth techniques render this type of superlattice especially appealing. An important step to implement the fabrication process has been achieved by producing crystalline superlattices without the need of growing the full layer stack by molecular beam epitaxy (MBE) but instead by growth of a strained bilayer of two materials which - upon release from the substrate - undergoes a rolling-up process^1, 2^. This leads to the formation of multilayer rolls, i.e. radial superlattices. A crucial point that determines and possibly hampers the potential applications of these tubes is the mechanical contact. It is determined by the adhesion between the individual semiconductor/semiconductor and semiconductor/oxide boundary surfaces. An imperfect mechanical contact can result for example into heat accumulation and thermal stress^13^ and influences the quality factor of optical cavities^14, 15^. Thus a detailed characterization of these structures is necessary to establish a better understanding of their mechanical properties including the adhesion between the individual layers as it was already investigated in other material systems by picosecond ultrasonics^16–19^.

Well established techniques to experimentally study the acoustic phonons and eigenmodes of nanostructures are static Raman and Brillouin spectroscopy which can provide access to the acoustic dynamics in the rolled-up superlattices^20–23^. However, the frequency resolution which has been achieved with these techniques on single structures, is very limited so far.

Here, we circumvent these limitations by using an optical pump-probe setup as a non-invasive characterization method^24^ which renders invasive and thus destructive methods, e.g., focused ion beam based characterization in combination with SEM imaging, unnecessary. This makes it possible to determine the distinct vibrational modes and to resolve single nanostructures spatially. We present a study of the acoustic phonon modes of the rolled-up multilayers and compare the results to planar multilayers grown by MBE. The experimental results are corroborated by calculations of the phonon mode spectra obtained via a transfer matrix method and the Rytov model.

We investigated 5 different types of samples. The layer structure can be divided into two different samples A and B. Furthermore, we distinguish the sample geometry in planar and rolled-up samples where sample A_p_ denotes the planar sample A and sample A_r_ the rolled-up sample A. For sample B the same notations are used. Sample A_5MBE_ consists of the same bilayer as sample A but has five repetitions of this bilayer, all grown by molecular beam epitaxy.

**Figure 1:**
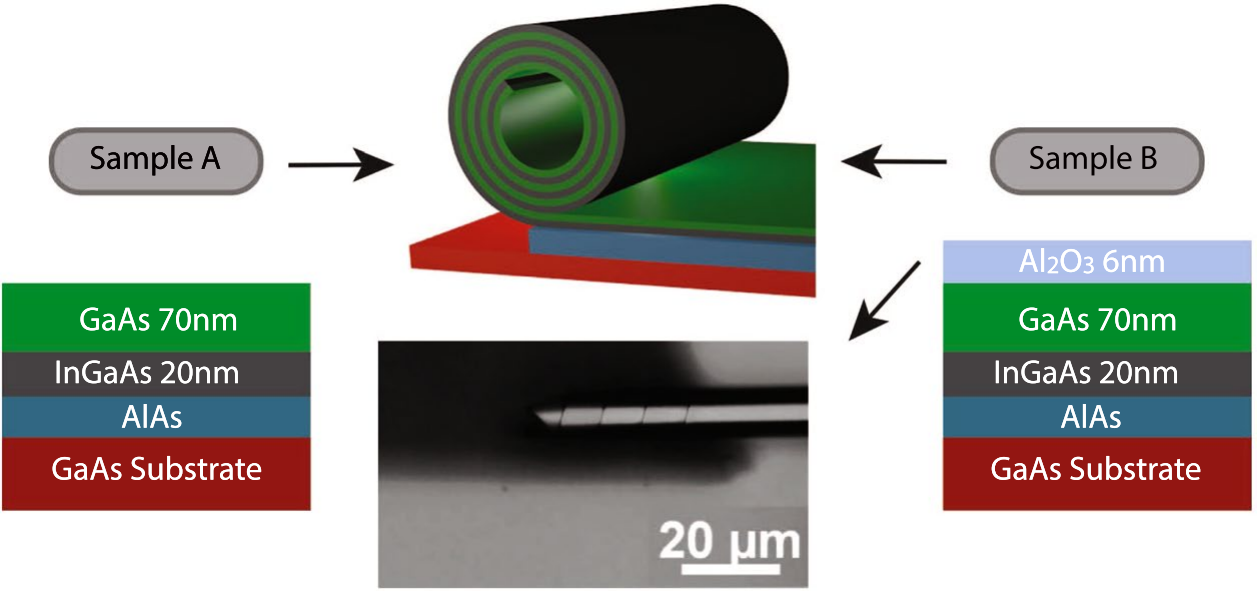
Sample structure with GaAs substrate and AlAs sacrificial layer and on top the rolled-up SL (middle part). Before the roll-up, sample A_p_ (left part) consists of a layer of GaAs and In_0.2_Ga_0.8_As while sample B_p_ (right part) has an additional layer of amorphous Al_2_O_3_ on top of the GaAs and In_0.2_Ga_0.8_As layers. The bottom part in the middle shows an image of sample B_r_ taken with an optical microscope. The number of windings increases from the left to the right from 1 to 5 which is controlled by the roll-up process.

The basic structures of the investigated samples A and B are shown schematically in Fig. 1. Molecular beam epitaxy is utilized to deposit two layers on a GaAs substrate forming the basis with a sacrificial layer of AlAs between substrate and layers. In sample A_p_ a layer of In_0.2_Ga_0.8_As (with the thickness *d*_*InGaAs*_ = 20 nm) and on top a layer of GaAs (*d*_*GaAs*_ = 70 nm) are grown on a sacrificial layer (see Fig. 1, left part). The In_0.2_Ga_0.8_As layer is compressively stressed through the MBE growth^1^. By removing the sacrificial layer the bilayer is released, which directly results in its roll-up into several windings (sample A_r_, Fig. 1, middle part). This roll-up process is driven by the interatomic forces of the In_0.2_Ga_0.8_As layer which expands the interatomic distances as soon as the AlAs is removed by HCl to release the inherent stress. This process in turn starts to stress the GaAs layer as its interatomic distance expands through the interface with the In_0.2_Ga_0.8_As layer. The number of windings determines the number of periods^8^ of the newly formed superlattice (SL) while the radius and the number of windings itself can be controlled by the thickness, strain and etching time of the epitaxial layers^2^. For the investigated samples in this paper the radii of the SL tubes are approximately 10 µm and the number of windings range from 1 to 5 resulting in a total tube wall thickness of up to 480 nm.

In comparison to the above described samples A_p_ and A_r_, sample B_p_ possesses an additional layer of amorphous Al_2_O_3_ (${d}_{A{l}_{2}{O}_{3}}$ = 6 nm) deposited by atomic layer deposition on top of the GaAs layer to avoid unintended side-rolling effects during release of the layer from the substrate as indicated in the right part of Fig. 1. In order to achieve a varying number of windings for sample B_r_ (Fig. 1, middle part), a trapezoidal shape is cut in the MBE grown layers, the Al_2_O_3_ is deposited and then the necessary trench for stress relief was cut at one side of the trapezoid. Therefore the number of windings varies in sample B_r_ depending on the position on the tube, with a maximum of layers at the centre. An optical microscope image of sample B_r_ is displayed in the middle part of Fig. 1, where the number of windings increases from the left (1 winding) to the right (5 windings). A fixed number like in sample A is obtained by a rectangular cut. For comparison and as a reference sample, a planar SL with 5 bilayers of GaAs (*d*_*GaAs*_ = 70 nm) and In_0.2_Ga_0.8_As (*d*_*InGaAs*_ = 20 nm) was additionally grown by MBE, sample A_5MBE_.

Optical excitation and detection of coherent acoustic phonons is realized by means of high-speed asynchronous optical sampling (ASOPS), which is a femtosecond time-resolved pump-probe technique^18^. Our setup consists of two mode-locked Ti:sapphire lasers, each with a repetition rate of approximately 800 MHz, which gives access to a temporal scan window of 1.2 ns with shorter than 100 fs temporal resolution. Pump and probe wavelengths are centred at 790 nm and 820 nm, respectively. The offset in the laser repetition rates is set to 5 kHz. A 50 × NIR microscope objective was used to focus the laser beam on the sample to a spot size of approximately 2 µm. A collinear setup in reflection geometry was used to perform the measurements. The pump pulse incident on the sample excites electron-hole pairs in the semiconductor layers resulting in an impulsive stress via the deformation potential. Hence, the acoustic eigenmodes of the structure are excited coherently. The acoustic phonons are monitored by the change in relative reflectivity by the delayed probe pulse. In this way we obtain direct access to the dynamics of coherent acoustic phonons in the system.

## Results and Discussion

In the following we will first present our results obtained from the as-grown, planar, unrolled samples A_p_ and B_p_ depicted in Fig. 1. We then move on to the MBE grown 5 period superlattice sample A_5MBE_ and finally discuss the coherent phonon dynamics in the rolled-up tubes for samples A_r_ and B_r_.

**Figure 2:**
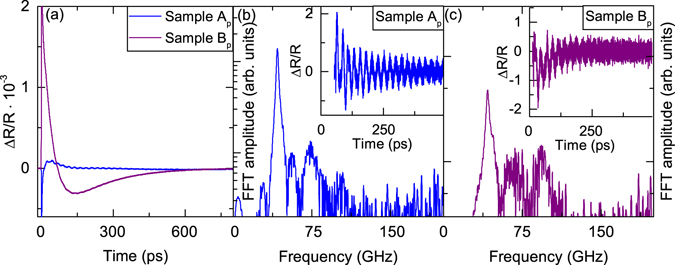
(**a**) Time-resolved reflectivity change of both planar samples A (blue) and B (purple). (**b**) FFT spectrum of the planar MBE grown bilayer (unrolled sample A as in the left part of Fig. 1). The inset shows the extracted time-resolved reflectivity change. (**c**) FFT spectrum of the planar MBE grown layers with an additional layer of Al_2_O_3_ (unrolled sample B as in the right part of Fig. 1). The inset shows the extracted time-resolved reflectivity change.

First, the planar MBE grown sample A_p_ is investigated. Upon excitation of the sample we obtain the time-resolved relative reflectivity change ΔR/R (see Fig. 2a). The transient shows a strong change in ΔR/R in the first picoseconds due to a fast excitation of the electron-hole pairs. Through electron-electron and electron-phonon scattering the excess energy is transferred to the lattice. We subtract the slowly decaying electronic and thermal background from the time domain data and extract the vibrational contributions of the phonons (insets in Fig. 2b,c). The respective Fourier transformations (FFTs) are shown in the main part of Fig. 2b,c. The FFT reveals distinct peaks at frequencies of 28 GHz, 42 GHz, 56 GHz, 73 GHz, and 103 GHz as shown in Fig. 2b. The most prominent frequency peak results from time-resolved Brillouin scattering in the GaAs substrate. This frequency appears at *f* = *2nv*_*GaAs*_ */λ* = 42 GHz, where *n* is the refractive index, *v*_*GaAs*_ = 4730 m/s the longitudinal sound velocity of bulk GaAs, for InGaAs *v*_*InGaAs*_ = 4550 m/s, and *λ* the wavelength of the probe laser pulse. We can unambiguously identify this peak after comparison to a reference measurement on a pure bulk GaAs substrate (for a comparison between the measurements on a part of the pure bulk GaAs substrate compared to the unrolled sample A_p_ see Supplementary Information A). The 28 GHz peak represents the combined thickness oscillation of both layers with *f* = *v/2d* where *v* = *((7/9)v*_*GaAs*_ + *(2/9)v*_*InGaAs*_ *)* is the combined weighted sound velocity of the layers and *d* the total bi-layer thickness. The other frequencies are the higher harmonics of the fundamental thickness oscillation (see Supplementary Information B).

Next, the FFT of the unrolled sample B_p_ is depicted in Fig. 2c. Distinct peaks can be seen at 42 GHz, 67 GHz and 93 GHz. Similar to sample A_p_ we find the frequency peak at 42 GHz which stems again from the time-resolved Brillouin scattering in the GaAs substrate. The other frequency peaks are a result of the combined oscillation of the layer system and higher harmonics (the measured frequency modes versus the mode number for the samples A_p_ and B_p_ on the substrate and for the samples as freestanding membrane are shown in the Supplementary Information B). The additionial Al_2_O_3_ layer, despite the small thickness, has a significant influence on the acoustic mode spectrum. The Brillouin oscillation is observed as long as the strain pulse travelling into the substrate is probed by the probe pulse, i.e. for a time given by t = (*αv*)^−1^ with *α* the absorption coefficient and *v* the sound velocity. The spectral width and lifetimes of the modes resulting from the eigenmodes of the top layers are determined by the rapid dissipation through coupling to the substrate. This allows us to distinguish the different layer systems by their eigenmode spectrum.

Before we have a closer look at the rolled-up structures, we investigate the planar superlattice sample A_5MBE_. Coherent acoustic phonons are excited and detected in the planar MBE grown superlattice with a repetition of 5 layers of GaAs and In_0.2_Ga_0.8_As.

**Figure 3:**
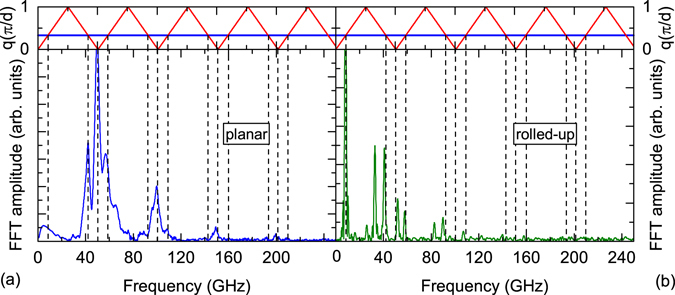
(**a**) The top part of the figure reveals the calculated back folded acoustic phonon dispersion relation (red line) with the observed 2*q*_*probe*_ modes at the intersection of the 2*q*_*probe*_ line (horizontal blue line) and the dispersion relation. The bottom part displays the FFT of sample A_5MBE_, the MBE grown SL with 5 layers of GaAs and In_0.2_Ga_0.8_As. Dashed lines indicate the eigenmode frequencies calculated by the Rytov model. (**b**) Similar to (**a**), but for the experimental results of the rolled-up SL of sample A_r_.

Figure 3a depicts the obtained mode frequencies (bottom part) and theoretical calculations (top part) of the acoustic phonon dispersion relation of sample A_5MBE_. The mode spectrum exhibits triplet structures which are characteristic for acoustic modes of a superlattice^25^. To gain a better quantitative understanding we calculate the acoustic phonon dispersion relation of a SL with two layers by means of the elastic continuum model according to Rytov (see Supplementary Information C for further details)^26, 27^.

Experimentally obtained frequencies show an excellent agreement to the modes of the calculated dispersion relation (dashed, vertical lines) despite the small number of periods of the SL^28^. Thus, we can conclude that 5 periods are in this case sufficient for acoustic SL behaviour^29^. We like to point out that the spectrum is independent of the position on the sample.

We now compare the just discussed results of a planar MBE grown superlattice to the superlattice structure obtained by the roll-up process, i.e. a rolled-up tube for the structure of sample A_r_. In Fig. 3b the FFT of the rolled-up SL is compared to the previously discussed sample A_5MBE_ in Fig. 3a. The distinct difference that can immediately be recognised is the absence of a triplet structure similar to the one observed in Fig. 3a. The dispersion relation and the expected mode positions are the same as and marked as before in Fig. 3a with dashed vertical lines to make this point more apparent. While some of the modes fit to the calculations we observe several modes which cannot be attributed for by this theoretical approach. The additional modes appear at frequencies *f* = 26 GHz, 32 GHz and 83 GHz. These modes can be explained by weak adhesion, we will come back to this point later on.

**Figure 4:**
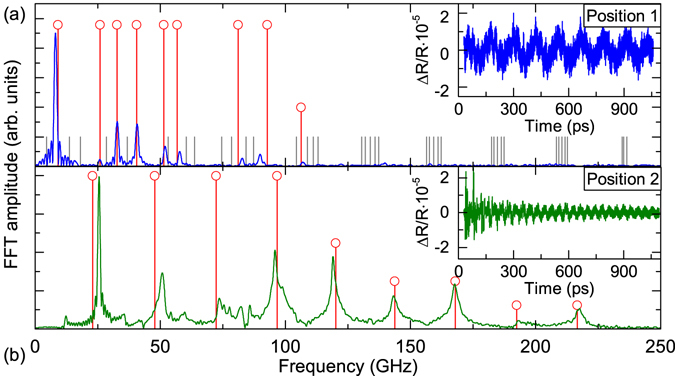
(**a**) The inset of the figure shows the extracted oscillations at position 1 of sample A_r_ (with a number of 5 windings). In the main part of the figure the respective FFT is shown. The vertical lines in the FFT spectrum are the frequency modes obtained from calculations based on the transfer matrix method. The corresponding sound velocities are listed in the main text. (**b**) Extracted oscillations and respective FFT with calculated frequency modes for position 2.

Furthermore, we like to emphasize that the tube structures also show distinct differences in their mode spectrum when different spatial positions are investigated. As an example we illustrate this behaviour in Fig. 4 (further details on the variation of measurements of sample A_r_ are shown in the Supplementary Information E and F). Figure 4a shows the time domain data and the respective FFT of position 1 as already presented in Fig. 3b. Figure 4b shows the data obtained on another position 2 on the same tube where a considerable increase in the mode spacing is observed. We will discuss these findings in the following.

So far our theoretical calculations have considered the system as an infinite superlattice. This approach worked well for the MBE grown superlattice but cannot account for our observations of the tube spectra. Therefore, we use a transfer matrix approach to calculate the acoustic eigenmode spectrum for a single bilayer up to five bilayers of GaAs and In_0.2_Ga_0.8_As in order to gain better insight into the acoustic properties of the tubes (for further details on the transfer matrix method see Supplementary Information D). The results of the calculations are presented as vertical lines in Fig. 4. The mode spectrum for position 2 (Fig. 4b) shows a very good agreement to our calculations of a single bilayer. Therefore, we can conclude that at this tube position the windings are not mechanically coupled and thus, we observe frequencies of the oscillation of a single bilayer of GaAs and In_0.2_Ga_0.8_As. The oscillations of the detached single bilayer contain the fundamental mode of the thickness oscillation which can be calculated with *f* = *v/2d* = 26 GHz and higher harmonics up to 220 GHz.

The eigenmode spectrum of position 1 is however not as straightforward to account for. Our first try assuming perfect adhesion between the 5 bilayers did not yield any results similar to the observed mode spectrum. Therefore, we introduced a massless spring to account for a non-perfect mechanical interface. The assumption of non-perfect interface adhesion is in this case reasonable as we already saw that a single tube exhibits areas where the roll-up process is not perfect but partly detached and we detect the characteristic mode spectrum of a single bilayer. The calculation presented for position 1 in Fig. 4a uses a massless spring between the individual bilayers assuming the simplest case with the same spring constant between each bilayer for the mechanical coupling between the neighbouring windings. A spring constant of *k* = 5 · 10^9^ kg/(m^2^ · s^2^) is obtained in this case which is several orders of magnitude smaller than typical metal/semiconductor contacts^30, 31^ (*k* = 6.32 · 10^18^ kg/(m^2^ · s^2^)) and epitaxially grown semiconductor layers. The modes that show the best coincidence with the measured spectrum are marked in red while all other modes are drawn in light grey for better visibility. We achieve a good qualitative agreement between the measured eigenmode spectrum and the calculated modes, therefore we conclude that the adhesion at this part of the tube is coupling all neighbouring windings and provides the spring constant as given above. If the different pairs of neighbouring windings are coupled by adhesion differently or some of them are not coupled by the adhesion at all, the mode spectrum shows a considerable more complicated pattern but can still be distinguished from areas where the two aforementioned cases of position 1 and 2 appear (see Supplementary Information F). We further assume that the main part of the signal stems from the upper part of the tube which basically resembles a type of freestanding membrane. This also accounts for the similar damping times of the two presented cases which are comparable in the time domain data in Fig. 4 due to the absence of a direct substrate coupling. In Fig. 4a the frequency of the lowest mode is significantly lower than the one in Fig. 4b and the spectrum in (a) even contains overall lower frequencies than in (b). Therefore, differences in amplitudes and in the damping times arise mostly from the slow decay of the long-living lowest frequency mode from (a). A quantitative evaluation of the damping times is beyond the scope of the current work.

Parts of the rolled-up tube where the layers are tightly attached can be distinguished from parts where they are loosely attached which agrees with our observations of non-uniform structures in tubes investigated by focused ion beam cutting and scanning electron microscopy (see Supplementary Information E).

The sound velocities used in the transfer matrix calculations deviate from the bulk velocities which we attribute to residual strain in the layers^21^. Although the layers are released from the sacrificial layer some residual stress remains. This effect has been taken into account in our calculations resulting in modified sound velocities. The best agreement between theory and experiment is obtained for the following sound velocities: for sample A_r_ they are found to change by a factor of 1.05 to *v*_InGaAs_ = 4778 m/s and with a factor of 0.89 to *v*_GaAs_ = 4210 m/s. As expected, the sound velocity in the In_0.2_Ga_0.8_As layer is increased as this layer is compressed, and *v*_*GaAs*_ is decreased as this layer is tensile stressed^32^. As can be seen in Fig. 4b, for the bilayer the key features of our measurements are well reproduced by our calculations. Minor deviations between theory and experiment can be due to the presence of an oxide layer on top of the sample^9, 33^.

In the following we present our results obtained from the tubes of sample B_r_ which exhibit increasing winding numbers (see Fig. 1 optical microscope image in the mid lower panel). Each area with a specific winding number was addressed and measured separately.

**Figure 5:**
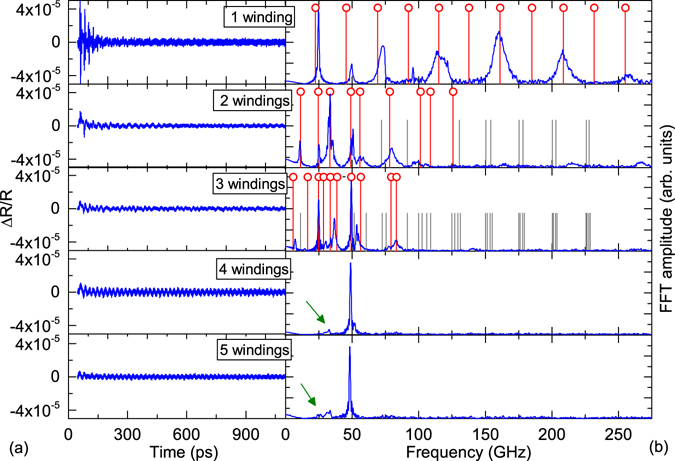
(**a**) The extracted oscillations of the reflectivity change of different parts of the tube (of sample B_r_) with a number of windings from 1 to 5 (from top to bottom) are presented. (**b**) The corresponding FFTs. The transition from the frequencies of a single oscillating bilayer towards modes of a multilayer is visible. In the FFT additionally to the experimental results also the calculations of the modes (vertical lines) based on a transfer matrix method with a massless spring in between the layers are presented. The green arrows mark the remaining acoustic contributions for the 4 and 5 windings sample for better visibility.

Figure 5a,b depict the extracted oscillations and the respective FFTs, sorted for different winding numbers. The time domain data in Fig. 5a already reveals a decrease in the acoustic signal response with increasing winding number. This becomes even more apparent in the frequency domain shown in Fig. 5b. We observe the characteristic eigenmode spectrum of the single triple layer which is similar to the results of the single bilayer in Fig. 4b. With increasing winding number the mode spacing decreases and we find a strong decrease in the acoustic signal amplitude. In fact for 4 and 5 windings the time resolved Brillouin contribution from the GaAs substrate dominates the signal completely and the tube modes nearly vanish (acoustic contributions are marked for better visibility with a green arrow). Before we discuss this intriguing point, the mode spectra up to three windings will be discussed in more detail. Following our earlier approach the eigenmode spectra are calculated including a massless spring connecting individual windings. For sample B, the sound velocities change due to the roll-up by a factor of 1.003 to *v*_InGaAs_ = 4564 m/s and with a factor of 0.9 to *v*_GaAs_ = 4273 m/s.

The vertical lines in Fig. 5b represent the calculated modes with a spring constant value of *k* = 5 × 10^9^ kg/(m^2^ · s^2^). We obtain again a good agreement between the measurement of the single three layer winding and our calculations where the spring constant does not have any influence on the calculation and we obtain the same results as without a spring. Small deviations may be caused due to our assumption of a planar geometry. Although the laser spot is considerably smaller than the tube width, the weak curvature may influence the mode spectrum slightly. We also find a good qualitative agreement between the calculations and the observed spectra for increasing winding numbers. The main features and the general trend of decreasing mode spacing with increasing winding number are reproduced.

A more detailed look at the sample structure is required for the discussion of the spectra for 4 and 5 windings. The main difference between this tube and the results of sample A_r_ presented in Fig. 4 where the acoustic contributions are still clearly visibly for a 5 windings tube, is the presence of the additional Al_2_O_3_ layer. With increasing winding numbers the possible contribution of this layer to the overall acoustic dissipation increases and can thus be responsible for a decrease of the detected phonon amplitudes. Another point which needs to be taken into account, is that the tubes form a kind of optical cavity where the upper and lower parts of the tube resemble mirrors with an air gap in between. This geometry can affect the detection process of coherent acoustics in the tubes and lead to a suppression of the detected phonons (for details see Supplementary Information G). Due to the high sensitivity of this effect on the air spacer thickness, i.e. the tubes diameter, a quantitative modelling of the detection process even for less winding numbers is strongly hindered.

Despite these current limitations for sample structure B_r_ we can unambiguously distinguish the winding numbers in these tubes up to three windings if the interface adhesion is sufficient for coupling of the individual windings while for sample structure A_r_ we can observe distinct acoustic features up to five windings.

## Conclusion

In conclusion, we have presented a detailed experimental and theoretical study of the acoustic phonon modes in rolled-up In_0.2_Ga_0.8_As/GaAs multilayer tubes. MBE grown planar multilayers were excited optically and the detected coherent acoustic phonon modes were analysed and compared to the modes in rolled-up multilayers with a varying number of windings. For the MBE grown 5 bilayer structure the obtained modes exhibit already the properties of a back-folded acoustic phonon dispersion relation of the SL and calculations with the Rytov model are in good agreement with the experimental results. Comparing it to the experimentally obtained results on two different layer systems of the rolled-up SL intriguing differences are detected. The Rytov model cannot account anymore for the obtained features due to imperfect adhesion between the individual windings of the tubes. We find a good qualitative agreement between a modelling approach based on transfer matrices including a massless spring which accounts for the interface adhesion and the observed eigenmode spectra. This allows us to distinguish areas with varying interface adhesion between the windings on individual tubes. We observe areas with sufficient interface adhesion to couple all of the individual tube windings as well as completely decoupled layers. A more complicated case arises when the interface adhesion only couples a part of the tube windings. These areas can be clearly identified but a detailed modelling is hindered due to the large number of free parameters. Furthermore the tubes exhibit areas where the detected signal amplitudes decrease drastically. This effect is attributed to the structure of the tubes that resembles an optical cavity and thus shows a strong sensitivity dependence on the inner tube radius. Our findings pose an important step towards the characterization of mechanical interface adhesion in nanoscaled systems. In particular the here investigated microtubes are a well suited system to study the mechanical coupling in multilayer systems with increasing complexity depending on the number of windings. These results are not only of general interest for all nanoscaled systems based on multilayer structures but are also crucial for the possible application of the studied microtubes in various fields in nanotechnology, e.g. microtube lasers, nanophotonics, thermoelectric devices and nanobiology, where structural homogeneity is crucial for reliable applications.

### Data availability statement

The datasets generated and analysed during the current study are available from the corresponding author on reasonable request.

## Electronic supplementary material

Supplementary Information
