# Supplementary material for: Interface Adhesion and Structural Characterization of Rolled-up GaAs/In0.2Ga0.8As Multilayer Tubes by Coherent Phonon Spectroscopy
Source: Sci Rep. 2017 Jul 14;7:5385. doi: 10.1038/s41598-017-05739-6 (PMC5511180; doi:10.1038/s41598-017-05739-6)
Supplement: Supplementary file 1 — Supplementary Information [file 41598_2017_5739_MOESM1_ESM.pdf]

Interface Adhesion and Structural Characterization of Rolled-up GaAs/$In_{0.2}Ga_{0.8}As$ Multilayer Tubes by Coherent Phonon Spectroscopy

D. Brick,[1,*] V. Engemaier,[2] Y. Guo,[1] M. Grossmann,[1] G. Li,[2] D. Grimm,[2] O.G. Schmidt,[2] M. Schubert,[1] V.E. Gusev,[3] M. Hettich,[1] and T. Dekorsy[1,4]

[1]*Department of Physics, University of Konstanz, 78464 Konstanz, Germany*

[2]*Institute for Integrative Nanosciences, IFW Dresden, Helmholtzstraße 20, 01069 Dresden, Germany*

[3]*LAUM, UMR-CNRS 6613, Université du Maine, Av. O. Messiaen, 72085 Le Mans, France*

[4]*Institute of Technical Physics, German Aerospace Center, Pfaffenwaldring 38-40, 70569 Stuttgart, Germany*

[*]*Corresponding author e-mail: delia.brick@uni-konstanz.de*

Supporting Information

A: Reference measurements on bulk GaAs

In Figure S1 the FFT of the unrolled sample A is compared to the FFT of pure bulk GaAs. The inset shows the time transient. The modes attributed to the thickness oscillation of the layers $In_{0.2}Ga_{0.8}As$ and GaAs vanish for the bulk substrate as expected while the mode at 42 GHz persists and can thus be unambiguously attributed to the time resolved Brillouin scattering in the GaAs substrate.

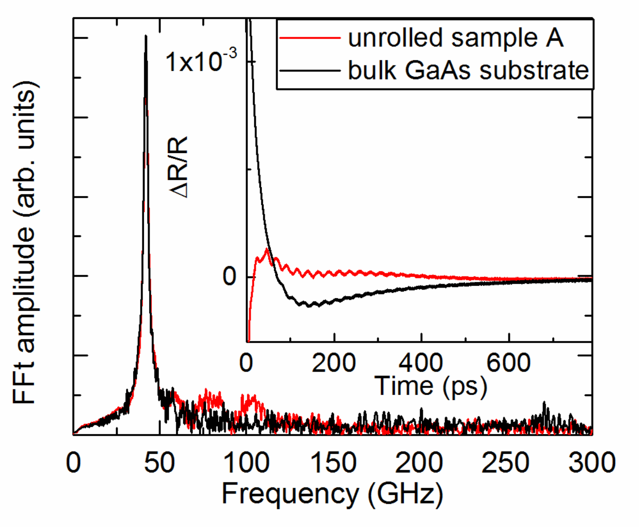

Figure S1: FFT of a part of the pure bulk GaAs substrate compared to the unrolled sample A_p. The inset shows the time transient.

B: Higher harmonics in the acoustic spectra of the unrolled samples A_p and B_p

For the samples A and B the frequency modes were obtained experimentally and plotted against the mode number for the two cases: A_p and B_p for the samples on a substrate and for A_r and B_r as a freestanding membrane. As expected we find a nearly linear relation with different slopes depending on the boundary conditions of the respective sample.[27]

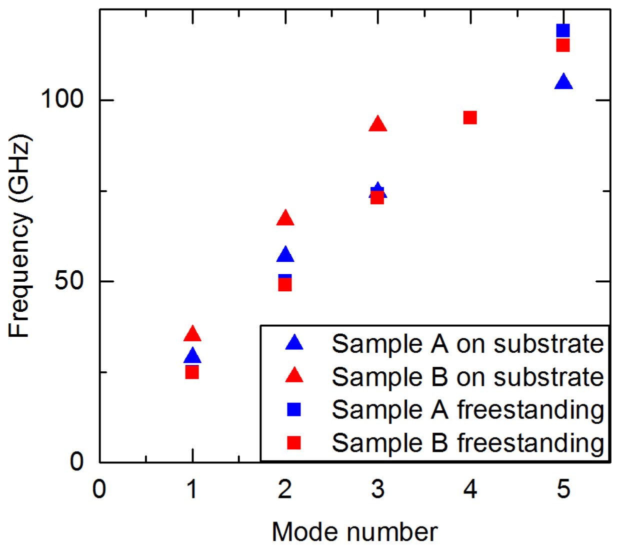

Figure S2: Frequency as a function of mode number for sample A and B on a substrate and as a freestanding membrane, obtained from experimental results.

C: Rytov model

To gain a better quantitative understanding we calculate the acoustic phonon dispersion relation of a SL with two layers X and Y by means of the elastic continuum model according to Rytov.[20] It is well known that in superlattices the electronic dispersion and the lattice-dynamical properties change considerably compared to the bulk material.[21] This is due to the repetitive structure of the superlattice as it introduces a new periodicity and hence a new boundary of the first Brillouin zone which is reduced to a mini-Brillouin zone with the boundary given by $q_B = \pi/d_{SL}$. Modes propagating in both material layers can be described by an average dispersion. In bulk materials the dispersion relation of acoustic phonons is linear with $\omega = vq$, whereas the dispersion relation of a SL can be approximated by the back folding of the bulk dispersion by reciprocal superlattice vectors.[20] While the generation of the acoustic phonons is restricted to the zone centre at $q = 0$, the detection is most sensitive at $q = 2q_{Probe} = 2\pi n/\lambda$, with q_{Probe} being the wave vector of the probe pulse.

The dispersion relation of a superlattice with two layers X and Y can be calculated by the elastic continuum model according to Rytov:[21]

$$\cos(\mathrm{qd}) = \cos\left(\omega\left(\frac{d_X}{v_X} + \frac{d_Y}{v_Y}\right)\right) - \frac{\epsilon^2}{2}\sin\left(\frac{\omega d_X}{v_X}\right)\sin\left(\frac{\omega d_Y}{v_Y}\right) \quad (1)$$

d_X, d_Y are the layer thicknesses and v_X, v_Y the speed of sound velocities, respectively. The degeneracy of the folded dispersion branches at the boundary and the centre of the Brillouin zone is eliminated by the correction term $\epsilon = \frac{\rho_Y \cdot v_Y - \rho_X \cdot v_X}{\sqrt{\rho_Y \cdot v_Y \cdot \rho_X \cdot v_X}}$, with the densities ρ_X and ρ_Y.

D: Acoustic transfer matrix method including massless spring

For the calculation of the frequency modes of the samples we use a transfer matrix method. A massless spring model is introduced between each winding to account for the imperfect adhesion. Here, we will derive the transfer

matrices for the case with a massless spring. More information on the transfer matrix formalism for acoustics are given for example in reference [34].

For a two layer system with layer A and B (see scheme) we obtain the transfer matrix T_{ab}:

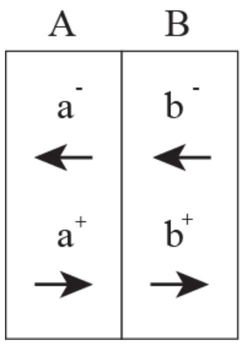

$$\frac{1}{2}\begin{pmatrix}(1+Z_{ab})e^{ik_a d_a} & (1-Z_{ab})e^{ik_a d_a} \\ (1-Z_{ab})e^{ik_a d_a} & (1+Z_{ab})e^{-ik_a d_a}\end{pmatrix}\begin{pmatrix}a^+ \\ a^-\end{pmatrix}=\begin{pmatrix}b^+ \\ b^-\end{pmatrix} \quad (2)$$

$$T_{ab}\begin{pmatrix}a^+ \\ a^-\end{pmatrix}=\begin{pmatrix}b^+ \\ b^-\end{pmatrix} \quad (3)$$

For a massless spring we have the boundary conditions: $\sigma_a = \sigma_b$ and $\sigma_a = k(u_b - u_a)$ this results into:

$\frac{1}{2}T_{ab}\begin{pmatrix}a^+ \\ a^-\end{pmatrix}=\begin{pmatrix}b^+ \\ b^-\end{pmatrix}$ where $T_{ab}=\begin{pmatrix}T_{11} & T_{12} \\ T_{21} & T_{22}\end{pmatrix}$ consists of $T_{11}=\left(\left(\frac{Z_a}{Z_b}\right)+\frac{i\omega Z_a}{k}+1\right)e^{ik_a d_a}$,

$T_{12}=\left(-Z_{ab}-\frac{i\omega Z_a}{k}+1\right)e^{-ik_a d_a}$, $T_{21}=\left(Z_{ab}-\frac{i\omega Z_a}{k}+1\right)e^{ik_a d_a}$, $T_{22}=\left(-Z_{ab}+\frac{i\omega Z_a}{k}+1\right)e^{-ik_a d_a}$.

E: Scanning electron microscopy image of rolled-up superlattice tube

The scanning electron microscopy image shows a cross section of sample A_r obtained by focused ion beam milling. The results obtained on different tubes (Figure S3) corroborate our findings of non-perfect interface adhesion as well as a non-uniform structural roll up behaviour.

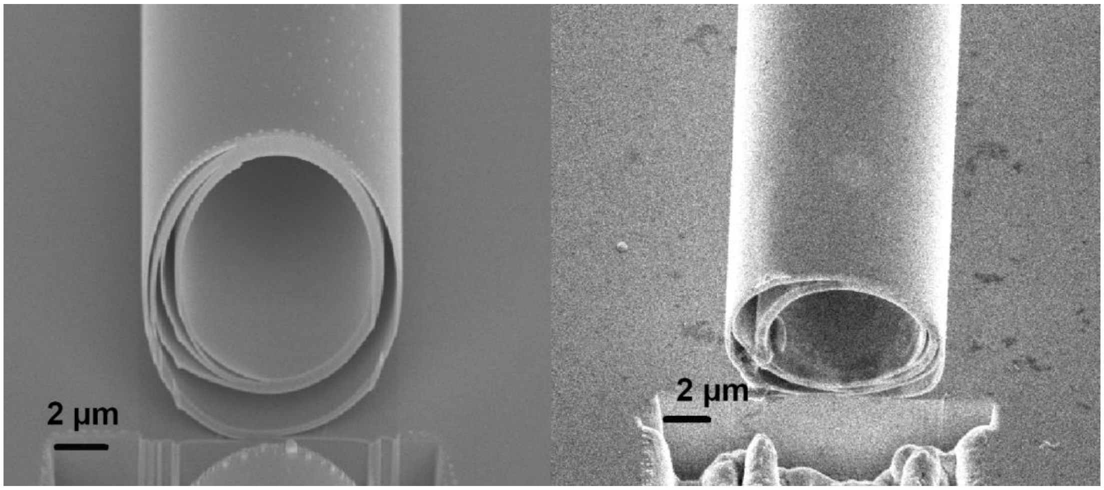

Figure S3: Scanning electron microscopy images of a cross section of sample A_r. Parts where the windings are closely attached and parts where they are not attached at all can be seen.

F: Acoustic mode spectra of partially coupled tube windings with sample structure A and spatially resolved scans along individual tubes

Different measurements of the modes of sample A_r with 5 windings are shown together with the modes calculated with the transfer matrix model including the massless spring using the same values for the calculations of sample A as pointed out in the main text (Figure S4 (a)). The system complexity for 5 windings including varying adhesion between individual windings results in a considerable number of free parameters which impedes a quantitative modelling. Depending on the coupling, the tubes undergo frequency shifts for a variation in the strength of the coupling or a superposition of mode spectra for sub-systems including one or more windings. Despite the complex mode spectra a grouping of the modes becomes apparent which is captured by the simulations for different winding numbers. This is indicated by the grey shaded areas which show the expected mode ranges given by the simulations for up to 5 windings in Figure S4 (a).

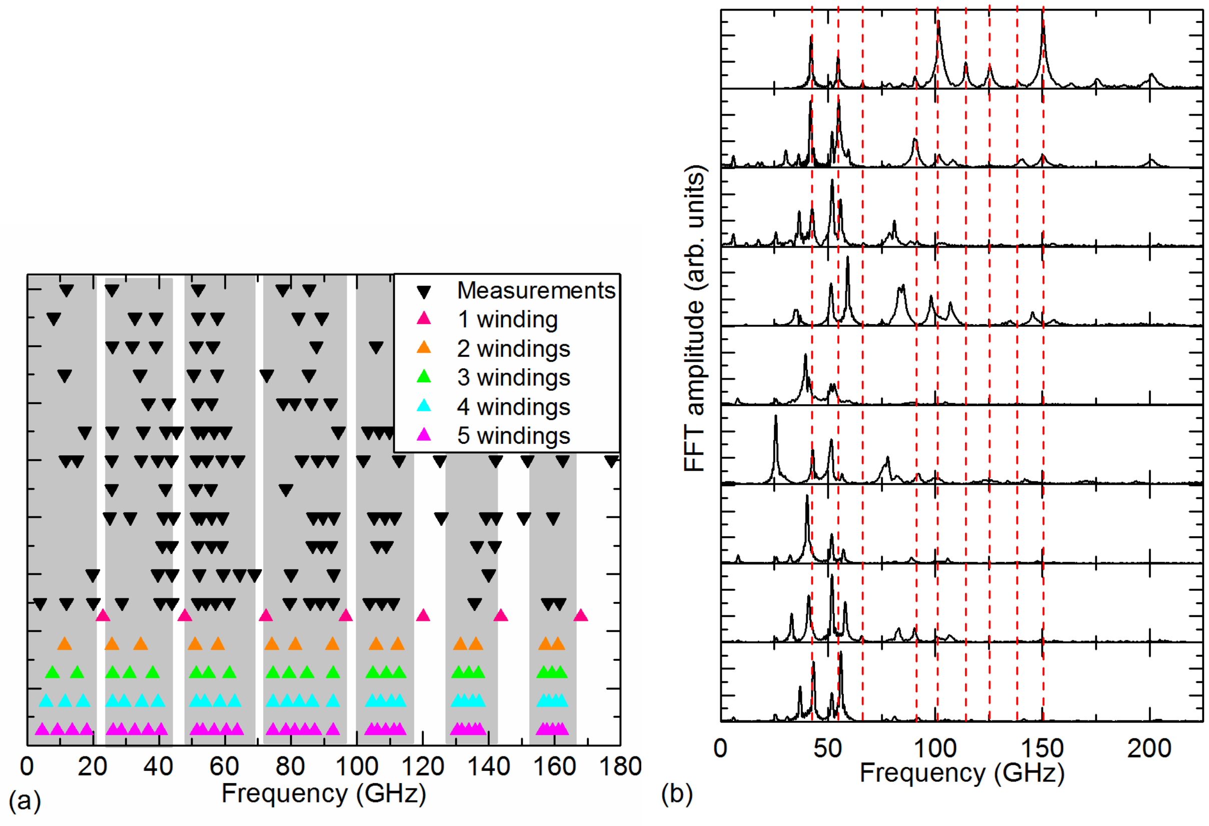

Figure S4: (a) Each row shows the peak frequencies of frequency spectrum of different measurements of sample A_r (black triangles) compared to calculations for 1 to 5 windings (coloured triangles). Areas of frequency groups are marked by a grey rectangle for better visibility. (b) FFT of scan along one tube of sample A_r. A clear shift of the frequencies is visible, the red lines are a guide to the eye for the shift of the frequencies.

In Figure S4 (b) the FFT of a scan along one tube of sample A_r is shown. A clear shift of the frequencies is visible. However, an unambiguous assignment of the modes to a certain amount of coupled windings is not possible in this case. This tube is one of the examples where different neighbour windings are coupled differently and there could be perfectly coupled, coupled by imperfect adhesion and completely decoupled neighbour windings. This by itself however is already an important information about the tubes structural quality and furthermore we can observe the distinct changes the mode spectra undergo along the tube which reveals a strongly inhomogeneous tube structure without sufficient winding adhesion.

G: Detection/ reflectivity as a function of wavelength

In Figure S5 (b) we can see that the reflectivity of the sample depends strongly on the air gap in the cavity of the

rolled-up tube, i.e. the inner tube radius (here shown for sample A_r, see Figure S5 (a)). For a probe wavelength of 820 nm the inner tube radius determines whether we are positioned on a slope or a minimum. In the first case high sensitivity is expected while changes in the reflectivity should be minimal for the second case. We also investigate this behaviour in more detail in Figure S5 (c) where we simulate the presence of coherent phonons by reflectivity calculations for several $In_{0.2}Ga_{0.8}As$ thicknesses. The slope at the nominal thickness of 20 nm is here a measure of the detection sensitivity and is strongly dependent on the inner tube radius. It is important to note that the actual phonon induced thickness change is far below the nanometre range. However, we decided to present the data in a broader thickness range in order to account for small deviations in the nominal layer thickness. The above discussion also holds true, e.g. if the nominal thickness is 21 nm.

(a)

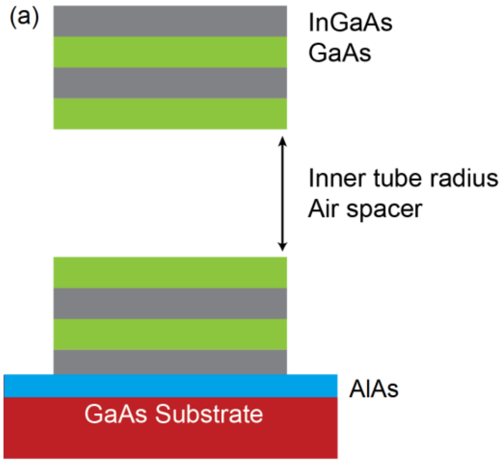

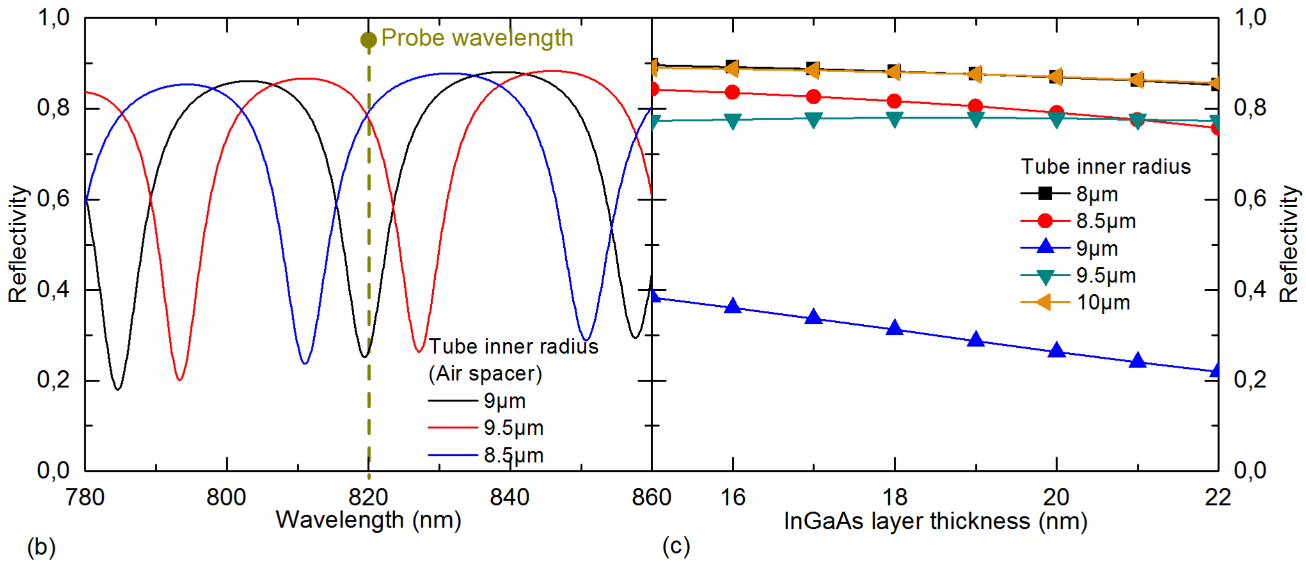

Figure S5: (a) scheme of layer stack of sample A_r. (b) the reflectivity as a function of the wavelength for three different tube inner radii (air spacer). (c) the reflectivity of sample A_r is shown, plotted against the $In_{0.2}Ga_{0.8}As$ layer thickness.

References

34. Tamura, S., Hurley, D. C. & Wolfe, J. P. Acoustic-phonon propagation in superlattices. Phys. Rev. B. 38, 1427 (1988).
